# Koopman Operator–Based Knowledge-Guided Reinforcement Learning for Safe Human–Robot Interaction

**DOI:** 10.3389/frobt.2022.779194

**Published:** 2022-06-16

**Authors:** Anirban Sinha, Yue Wang

**Affiliations:** Mechanical Engineering Department, Clemson University, Clemson, SC, United States

**Keywords:** deep reinforcement learning (DRL), deep Q network (DQN), Koopman operator, learning from demonstration, human knowledge representation

## Abstract

We developed a novel framework for deep reinforcement learning (DRL) algorithms in task constrained path generation problems of robotic manipulators leveraging human demonstrated trajectories. The main contribution of this article is to design a reward function that can be used with generic reinforcement learning algorithms by utilizing the Koopman operator theory to build a human intent model from the human demonstrated trajectories. In order to ensure that the developed reward function produces the correct reward, the demonstrated trajectories are further used to create a trust domain within which the Koopman operator–based human intent prediction is considered. Otherwise, the proposed algorithm asks for human feedback to receive rewards. The designed reward function is incorporated inside the deep Q-learning (DQN) framework, which results in a modified DQN algorithm. The effectiveness of the proposed learning algorithm is demonstrated using a simulated robotic arm to learn the paths for constrained end-effector motion and considering the safety of the human in the surroundings of the robot.

## 1 Introduction

In robot motion planning, we are interested in finding a path from the start to the goal such that at all the intermediate points, the robot is collision free. Motion planning for robots has a rich literature and has been very successful (LaValle, 2006). In imitation learning (IL) Schaal (1999), the robot path is generated by utilizing human demonstrated trajectories, which have been proven to be effective for generating paths for complex tasks. The use of reinforcement learning (RL), along with the demonstrated data, has emerged effective as it allows the optimal policy to be learned by interacting with the planning environments (Finn et al., 2016). However, one of the major challenges with this approach is obtaining the appropriate reward function for the RL agent. Furthermore, the demonstrated data used in these algorithms are expected to contain rich observation data. These algorithms are often computationally expensive and take a long time to learn the optimal trajectories.

**FIGURE 1:**
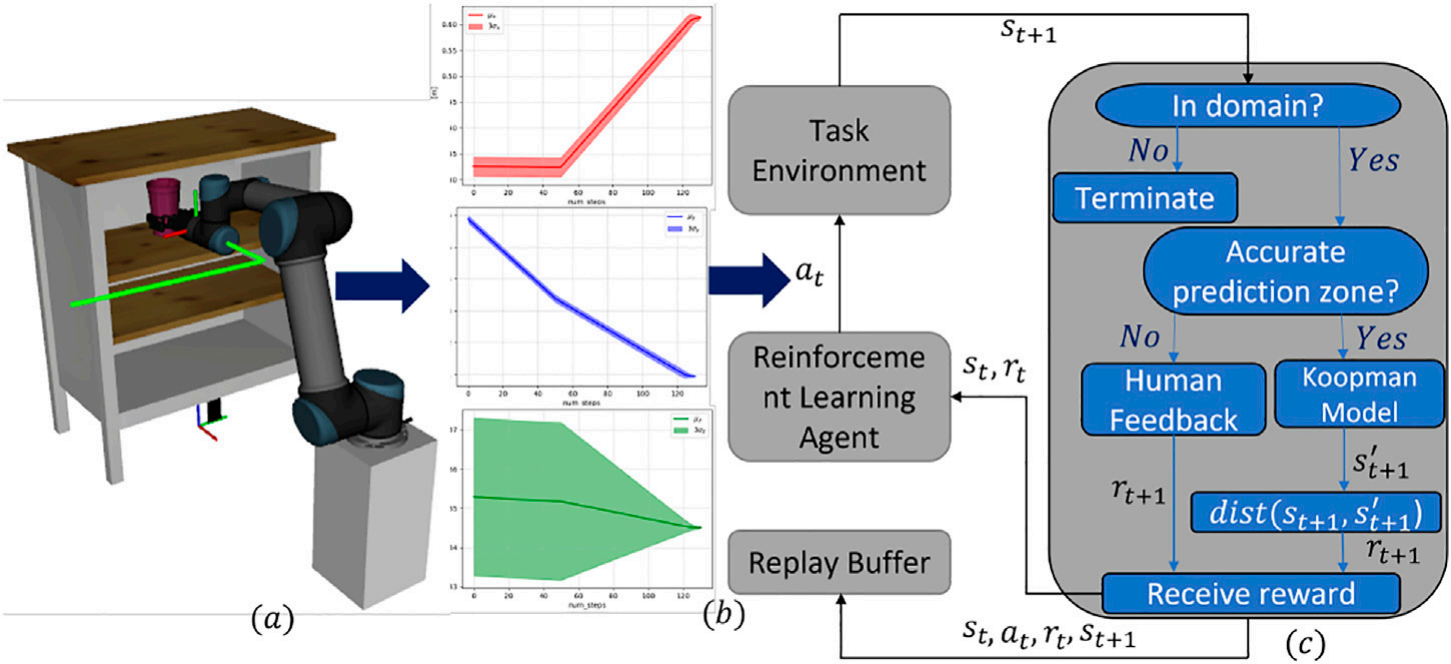
Koopman operator–based knowledge-guided RL framework: **(A)** A simulated UR5 robot is executing a demonstrated trajectory to perform *putting a cup in a shelf* task. The demonstrated path is shown using a green firm line. **(B)** Mean and standard deviations of the components of the position vectors, i.e., *x*, *y*, *z*, of all the way points in the demonstrated trajectories. **(C)** Schematic diagram of the training loop of the RL agent using the proposed methodology.

In this article, we propose a novel knowledge-guided deep reinforcement learning (DRL) framework to learn path planning from human demonstrated motion. The Koopman operator is used to develop the representation of human intent from the demonstrated trajectories for some tasks, which are then used to design the reward function of the RL-based autonomous planning agent. During the learning phase of the DRL agent, at each step, the state achieved by the agent after taking an action is compared to the human-preferred state predicted by the Koopman model of human intent to decide the reward that the DRL agent would receive. However, the Koopman operator model of the human intent would only be effective in the domain where the demonstrated data points are available, resulting in wrong or ambiguous state prediction of the human intent, which makes designing the appropriate reward function challenging. In order to alleviate this issue, the demonstrated trajectories are further used to generate a trust region in which the Koopman model prediction can be relied upon to provide the DRL agent an appropriate reward. To the best of our knowledge, we are the first to use the Koopman operator to design the reward function for the DRL agent. The reason Koopman operator is effective in modeling nonlinear data is that it uses several nonlinear basis functions to capture the underlying nonlinear behavior of the data unlike the linear regression model. Figure 1 outlines the end-to-end workflow of learning to plan a path from demonstrated trajectories by an RL agent utilizing the Koopman operator–based human intent model to design the reward function.

Furthermore, to facilitate the DRL agent to explore regions where no demonstrated data are present or the Koopman model's stated predictions are inaccurate, a provision is made to accept human feedback in such states in the proposed learning framework. We assume that the human feedback is always optimal, and the human expert knows what the optimal state is at any stage of the learning phase of the DRL agent. We have used human feedback in terms of a fixed numeric positive reward value for being at the right state (Griffith et al., 2013), otherwise a fixed negative reward is received. This simple pair of human reward values will reduce the effort of the human expert. Furthermore, we chose numeric rewards as human feedback, as they are relatively easier to provide by a human expert instead of providing a correct state or action feedback. Since the DRL agent only queries for the human feedback when there is no demonstrated data or when the Koopman model of human intent is inaccurate, the number of queries is significantly lesser than the feedback asked for after every iteration Christiano et al. (2017).

We have presented two examples of learning human preferred trajectories using the proposed algorithm which resulted in successful learning of the expert's trajectories. The second example also signifies the potential of using the proposed learning algorithm in achieving human preferred trajectories where safety of the nearby human is essential.

Our key contribution in this article is the modeling of the human intent using the Koopman operator theory and making use of that intent model to design the reward function for an autonomous planning agent which is an RL agent. Furthermore, human demonstrated trajectories are utilized to obtain a trust domain within which the Koopman model's predictions are considered. Also, the proposed algorithm asks for human feedback occasionally, and the human expert has to provide a fixed positive or negative reward which is relatively less cumbersome than other feedback mechanisms used in the RL literature. For details, please see Wirth et al. (2017) and the references therein. The efficacy of the proposed RL algorithm is demonstrated with two examples. In both the examples, the proposed algorithm is utilized to learn human demonstrated trajectories to the six degrees of freedom of a universal robot. It is shown that the Koopman operator–based reward function for the RL agents can effectively learn the human expert's trajectories in situations where the motions of the robot end-effector are geometrically constrained (please see the example in Section 5.1). In the second example, the proposed algorithm is utilized to learn a human demonstrated path where the safety of the human subject in the surroundings takes priority. Finally, we have presented a modified DQN learning framework with a Koopman model of human intent. We have chosen DQN agent to leverage the simplicity of the algorithm such that the new modifications are incorporated in it easily. We could have used any other reward-based RL algorithms and our proposed modifications would have been equally valid.

The proposed learning method can be treated as an alternative approach to the *inverse reinforcement learning* (IRL) and *generative adversarial imitation learning* (GAIL) for imitation learning problems. Unlike IRL and GAIL, our method does not need a reward function to be learned, instead we show that a fixed set of reward values can be used to learn from the demonstrated trajectories with some simple modifications in the DQN algorithm.

The article is organized as follows. The next section provides the works related to the algorithm presented in this article. In Section 3, we state the problem mathematically and propose knowledge-guided DQN with the Koopman operator-based reward function. In Section 4, we describe the proposed methodology in algorithm format. Section 5 presents two case studies with learning from human preferred trajectories using the proposed algorithm. Finally, we conclude the work in Section 6 with the discussion on the potential direction to pursue this work in the future.

## 2 Related Work

To plan motion for robotic arms using any learning techniques, the main objective is to identify the right action given the observations of the robots' states and their surrounding environment. In many robotic applications, the planning agent is desired to take actions that a human expert would take, given the same observations. This leaning paradigm is known as *imitation learning* (IL). For any realistic robotic tasks, the size of the observation space becomes large and learning to take human preferred actions in that observation space becomes an overly challenging task. However, it has been proved by the researchers that incorporation of human demonstrations in the learning algorithm is not only effective but also helpful in reducing the learning time (Bakker and Kuniyoshi, 1996; Schaal et al., 1997; Schaal, 1999; Billard and Matarić, 2001).

Traditionally in IL, the demonstrated trajectories are used as data points consisting of state–action pairs to train a deep neural network-based agent with the assumption that each data point comes from a *Markov decision process* (MDP) (Ross and Bagnell, 2010; Ross et al., 2011). The problem with Ross et al. (2011) is that the agent only learns how to mimic experts' behavior but fails to take the right actions where no demonstrated data are present and cannot learn a better policy than the experts. Essentially, Ross et al. (2011) does not consider the cost of making a right or wrong action. In Ross and Bagnell (2014), an RL perspective of the IL has been introduced by considering the cost (cost-to-go) of an action from a given state as a *Q*(***s***, *a*) value to the RL algorithm. Hester et al. (2018) used deep *Q* learning to learn from small sets of demonstrations by combining temporal difference updates with supervised classification of the demonstrators' actions. The work in Hester et al. (2018) assumes that the demonstration data provide both states and actions which are true in many gaming environments, but when a human demonstrates for a robot, he/she only provides the states as end-effector pose, and no direct actions are provided. It further assumes that the demonstrated data are noise free, hence they completely represent the expert's behavior. Furthermore, unlike gaming environments, while working with real robots, there is no reward function that is defined which is essential for the success of the RL algorithms.

*Behavioral cloning from observation* (BCO) (Torabi et al., 2018) considers only the states as the experts' demonstration, and the corresponding underlying actions are inferred from a learned dynamics model of the robot and then IL is performed with the states and recovered actions. A linear regression model is developed using the collected data from a human prosthesis device that represents a knowledge base, which is then used to guide a Q-learning process in Gao et al. (2020). However, a linear regression model of the state–action pair over the entire demonstration domain can limit the use of the underlying knowledge.

In IL, the field of *active learning* allows the learning agent to query the human expert for optimal state/action from a given state where there is no demonstration data, and this is used to improve the current policy. This technique has been proved to be data efficient and learns the optimal policy quicker. In these approaches, the learned agent executes the policy and asks for new samples from the human in places where the agent does not feel confident Chernova and Veloso (2008). In Jin et al. (2020), a parametric reward function that is a representative of the experts' intention is learnt from the sparsely demonstrated way points. However, the way points are given with respect to a certain time instance and selection of such way points also impacts the success in learning the parametric reward function.

Success of the RL algorithms is vastly dependent on the reward function. However, in many situations the appropriate reward function is unknown to the agent or may be partially known. In inverse RL (IRL) (Ng and Russell, 2000; Ziebart et al., 2008), the reward function is developed simultaneously with the learning process with the agent. Although IRL methods have been proven to be effective in learning reward functions for a variety of the problems, they are computationally heavy and need a huge number of iterations before the learning processes are completed. Further use of IRL to learning control policy from the human demonstrations with unknown robot dynamics can be found in Finn et al. (2016).

The work in this article is targeted toward designing a simple but effective reward function for the RL algorithms to learn paths from human demonstrations. In this context, we have developed the Koopman operator-based human knowledge representation from human demonstrated trajectories. Unlike IRL techniques, our reward function does not change during the learning process, which makes the DRL agent to learn faster for certain kinds of robotics tasks as presented in Section 5. Furthermore, the demonstrated trajectories required for the proposed algorithm merely contain the end-effector poses and the learning agent recovers the underlying correct actions if the kinematics of the robot is known. A similar approach has also been used by Niekum et al. (2015). Furthermore, unlike in Gao et al. (2020) where human knowledge has been developed using a linear regression model, in our work the use of the Koopman operator to develop human knowledge representation can accommodate nonlinearity in the human intent model. Our work is partially related to the idea of using DQN for IL as introduced in Hester et al. (2018). However, we have designed a task-specific reward function using the Koopman theory without assuming that a reward function is available to the learning agent. While in Hester et al. (2018), the reward function was assumed to be given. *Soft Q imitation learning* (SQIL) has been proposed in Reddy et al. (2019) where it is shown that a simple Q learning–based agent with fixed-type reward values can be used to solve IL which outperformed GAIL. Our proposed method also uses fixed reward type of reward values, but as to which fixed reward value the agent would receive is decided by the Koopman operator–based human intent model's prediction. We characterize our proposed method as an alternative to Reddy et al. (2019) and further confirm the idea that simple Q learning with a fixed type of reward can be used for IL problems. Furthermore, Abraham et al. (2017) and Broad et al. (2020) used the Koopman theory to obtain a data-driven linearized model of a system, which was then used to obtain a model-based controller. However, our work uses the Koopman theory to obtain human intent model from the demonstrated trajectories to predict the human preferred state from a state where the RL agent arrived during exploration of the environment.

## 3 Knowledge-Guided Reinforcement Learning

In this article, we are interested in developing a knowledge-guided RL algorithm to obtain polices by learning from the state-only observation sequences to accomplish a specific task. Our goal is to utilize the demonstrated state observation sequences as a knowledge base, which we further use to predict the desired state of the learning agent as compared to the actual state that the agent has arrived at after taking an action to decide the reward value that the agent would receive for that action.

Let us first define some of the notations that we will be using throughout the article. We denote the state observation sequences or trajectories as a set $D_{\text{demo}}=\left\{T_{1},T_{2},\ldots ,T_{n}\right\}$ where each ***T***_***i***_, *i* ∈ 1, …. *n* represents a full demonstrated trajectory and *n* is the number of demonstrated trajectories. Each ***T***_***i***_ is composed of a sequence of the end-effector states of a manipulator, i.e., ***T***_***i***_ = ***s***_**1**_, ***s***_**2**_, *…*, ***s***_***m***_, where each ***s***_***j***_, *j* ∈ 1, *…*, *m* is a state of the robot end-effector and *m* is the length of a trajectory. The state transition pair (***s***_***j***_, ***s***_***j*** +**1**_) for any given value of *j* carries the signature of the feature of the transition for the step number *j*. Our objective is to utilize this state transition information of the demonstrated trajectories to create a knowledge base that will be representative of the human expert's intent while demonstrating the trajectories. We assume that the human demonstrated trajectories are the optimal trajectories that the DRL agent tries to imitate. The DRL agent finds the policy $\pi_{\theta }:S\to A$ that maps an input state ***s*_*j*_** and recovers the hidden action *a*_*j*_ such that ***F*** (***s***_***j***_, *a*_*j*_) = ***s***_***j*** **+1**_ will be close to the state that the human expert would prefer. The notations S and A represent the set of all possible end-effector states of the robot and the set of all possible actions the DRL agent can take. ***F*** (***s***_***j***_, *a*_*j*_) is the state transition function that takes ***s*_*j*_** and *a*_*j*_ as input and returns the next state ***s***_***j*** +1_ of the robot.

### 3.1 Deep Q Network as Learning Agent

We pose the IL problem as an MDP which is represented by a 5-tuple <S,A,F,r,γ> , where *r*(*a*_*j*_, ***s*_*j*_**) is the immediate reward function that the agent receives by taking action *a*_*j*_ from state ***s*_*j*_**, and *γ* is the discount factor. The DRL agent explores different actions from A to learn to maximize the expected discounted reward. In this context, *Q* ^*π*^(***s***, *a*) represents the expected total reward that the agent can get by following the policy *π*. The *Q* ^*π*^(***s***, *a*) can be expressed as *Bellman equation* given as follows:

$$Q^{\pi }\left(s_{t},a_{t}\right)=E\left[r\left(s_{t},a_{t}\right)+\gamma \underset{a}{\max}Q^{\pi }\left(s_{t+1},a_{t+1}\right)\left|\right.|s_{t},a_{t}\right]$$ (1)

Then, an optimal policy *π*∗ is defined as

$$\pi^{*}\left(s\right)=\underset{a}{\text{argmax}}Q^{\pi }\left(s,a\right)$$ (2)

A deep Q network approximates *Q*-function with a neural network. In this article, we particularly used double DQN (Van Hasselt et al., 2016), where a target network is used to find the loss between the current and desired prediction of the *Q* values. This loss is then used to update the weights of the neural network representing the agent. The squared loss of the double DQN is defined as follows:

$$loss={\left(r\left(s_{t},a_{t}\right)+\gamma \underset{a}{\max}Q\left(s_{t+1},a_{t+1}\right)-Q\left(s_{t},a_{t}\right)\right)}^{2}$$ (3)

In the case of DQN with *experience replay*, all the experiences (state, action, and reward) while exploring different actions are stored in a buffer storage and a batch (collection of the stored experiences) is selected by randomly sampling experiences from the buffer to train the deep Q network. Furthermore, human demonstrated trajectories are used to obtain a model of human expert's intention. In this article, we have proposed the Koopman operator–based human knowledge representation. The knowledge representation is a statistical function, say F , which takes the current state of the DRL agent (***s*_*t*_**) as input and returns the predicted next state ***s***_***t*** +1_. This predicted state can then be compared with the actual next state of the DQN agent to make the decision on the reward that the agent should receive. To be more specific, to train the DQN agent, the value of the *r* term in the back-propagated loss in Eq. 3 is decided using the Koopman model representation of human intent. In the next section and the following ones, we will present briefly the Koopman operator theory and how that is used to obtain the reward of the DRL agent.

### 3.2 Koopman Operator–Based Human Knowledge Representation

The Koopman operator (Henrion et al., 2016) has been traditionally a data-driven method used to obtain a linear model of a nonlinear dynamic system. Let us consider a discrete-time dynamical system as

$$x_{k+1}=f\left(x_{k}\right)$$ (4)

where ***f*** (⋅) is a nonlinear function and $x_{k},x_{k+1}\in R^{l}$ indicates an *l* dimensional state vector of a time-varying system at time step *k* and *k* + 1, respectively. Suppose, we are given a vector-valued function ***g*** (⋅) such that ***g***: ***x***_***k***_ → ***y***_*k*_ where $y_{k}\in R^{l^{\prime }}$ and *l*′ > *l*. That is, the function *g* simply lifts the lower dimensional vector ***x*_*k*_** to a higher dimensional vector ***y*_*k*_**. In the higher dimensional space of dimension *l*′, according to the Koopman theory, there exists a linear operator $K\in R^{l^{\prime }\times l^{\prime }}$ , which maps ***y*_*k*_** to ***y***_***k*** +1_ as follows:

$$y_{k+1}\approx Ky_{k}$$ (5)

$$\;\Rightarrow \;g\left(x_{k+1}\right)\approx Kg\left(x_{k}\right)=g\left(f\left(x_{k}\right)\right)$$ (6)

Please note that, in general, the function ***g*** (⋅) is unknown. However, there are several data-driven (Lusch et al., 2018) and model-based (Abraham et al., 2017) techniques to approximate ***g*** (⋅). Unless ***g*** (⋅) is an infinite dimensional vector, there will be an approximation error or residual error. Using the residual error as *r*_*e*_, the approximate linear dynamics in finite dimensional lifted space is described as

$$g\left(x_{k+1}\right)=Kg\left(x_{k}\right)+r_{e}$$ (7)

We are interested in finding ***K*** such that the value of *r*_*e*_ is minimum. In this research, we have adopted the least-square method to compute ***K*** as given by Williams et al. (2015).

Suppose, we are given *M* data points of one trajectory or multiple trajectories (not necessarily in order), then the total residual due to linear approximation is obtained as

$$R=\overset{M-1}{\underset{m=1}{\sum }}r_{e_{m}}=\overset{M-1}{\underset{m=1}{\sum }}|\left(g\left(x_{m+1}\right)-Kg\left(x_{m}\right)\right)|$$ (8)

Then, the least-square optimization problem can be formulated as

$$K^{*}=\underset{K}{\mathrm{argmin}}\frac{1}{2}\overset{M-1}{\underset{m=1}{\sum }}|\left(g\left(x_{m+1}\right)-Kg\left(x_{m}\right)\right){|}^{2}=\underset{K}{\mathrm{argmin}}\frac{1}{2}\overset{M-1}{\underset{m=1}{\sum }}|r_{em}{|}^{2}$$ (9)

where K* is the optimal ***K*** for which the residual *R* will be minimum. It can be shown that the least square solution of the optimization problem in Eq. 9 will be (Abraham and Murphey, 2019)

$$K^{*}=AG^{†}$$ (10)

where the operator † represents pseudo-inverse of a matrix and

$$A=\frac{1}{M}\overset{M-1}{\underset{m=1}{\sum }}g\left(x_{m+1}\right)g{\left(x_{m}\right)}^{T}$$ (11)

$$G=\frac{1}{M}\overset{M-1}{\underset{m=1}{\sum }}g\left(x_{m}\right)g{\left(x_{m}\right)}^{T}$$ (12)

### 3.3 Designing Reward Function for the Deep Q-Learning Agent

In this work, we have used the Koopman operator to represent human knowledge base. More specifically, we have identified the Koopman operator ***K***_***p***_ based on human demonstrated trajectories such that the given current end-effector state ***s*_*t*_** and the human preferred end-effector state can be obtained as

$$g\left(s_{t+1}^{\prime }\right)=K_{p}g\left(s_{t}\right)$$ (13)

$$s_{t+1}^{\prime }=g^{-1}\left(g\left(s_{t+1}^{\prime }\right)\right)$$ (14)

where ***g*** is the same as defined in Eq. 6 and ***g* ^−1^** (⋅) is the inverse transformation of ***g*** (⋅). Once the desired end-effector pose $s_{t+1}^{\prime }$ is predicted by the Koopman operator model of human intent, then we can compare it with the state that the DRL agent reached after taking an action by the DQN agent. Let ***s*_*t*+1_** be the state that the agent has reached after taking action *a*_*t*_ from state ***s*_*t*_**. If ***s*_*t*+1_** is sufficiently close to $s_{t+1}^{\prime }$ , then the agent receives a positive reward, otherwise the agent receives a negative reward. Since both ***s*_*t*+1_** and $s_{t+1}^{\prime }\in \mathrm{SE}\left(3\right)$ (special Euclidean Group which represents all the possible poses of a rigid body moving in space) and there is no bi-invariant metric that can be defined on SE(3), we compared the translation and rotation parts of ***s***, ***s*′** separately. Let $p_{t+1}\in R^{3}$ and ***R***_***t*** **+1**_ ∈ *SO*(3) (special orthogonal group of dimension 3) be the position and orientation components of the agent's state ***s*_*t*+1_**, respectively. Similarly, let $p_{t+1}^{\prime }\in R^{3}$ and $R_{t+1}^{\prime }\in SO\left(3\right)$ be the position and orientation components of the predicted state $s_{t+1}^{\prime }$ , respectively. Then, the distance metrics that we choose for position ***p*_*dist*_** and orientation ***R*_*dist*_** distances are defined as follows:

$$p_{\mathrm{dist}}=\|p_{t+1}-p_{t+1}^{\prime }{\|}_{2}$$ (15)

$$q_{\mathrm{dist}}=\|\log\left(R_{t+1}R_{t+1}^{T\prime }\right){\|}_{2}$$ (16)

where ‖ ⋅‖_2_ represents 2-norm of a vector. Another advantage of using the Koopman operator to represent human knowledge base is that if at any step the DRL agent takes a wrong action from a given state, it can step back and explore the other possible actions from that state and compare the resulting state with the state suggested by the knowledge base. The agent can then know from that state what are the right and wrong actions to take and can store both these experiences to utilize them during experience replay.

### 3.4 Trust Domain for the Koopman Prediction

In RL, appropriate reward signal plays an important role in the success of the learning process. In our case, this is hinged at the correct prediction of the human intended state of the agent $s_{t+1}^{\prime }$ as given in Eq. 13. Since ***K*_*p*_** is calculated from the states in the demonstrated data set $D_{\text{demo}}$ , prediction of $s_{t+1}^{\prime }$ could be inaccurate if ***s*_*t*_** does not belong to the domain of $D_{\text{demo}}$ . In order to alleviate this issue, we identified the distributions of the components of the positions and orientations for each step of the demonstrated trajectories. That way, we obtain two sets of vectors, one for the means and the other for the standard deviations for each component of the position and orientation element, where the lengths of the vectors are the maximum step of all the demonstrated trajectories. All the parameters of the distributions together define the trust domain. Any state of the RL agent that lies inside this domain can be used to determine the next state using the Koopman operator.

Let us denote $s_{t}^{i}=\left[x_{t}^{i},y_{t}^{i},z_{t}^{i},\alpha_{t}^{i},\beta_{t}^{i},\gamma_{t}^{i}\right]$ as the state vector of the *i*th demonstrated trajectory at step *t*; then, we are interested in getting vectors $\mu_{l}\in R^{m^{\prime }}$ , $\sigma_{l}\in R^{m^{\prime }}$ , where *m*′ is the final step number of the demonstrated trajectories and *l* ∈ {*x*, *y*, *z*, *α*, *β*, *γ*}. The *t*th elements of ***μ*_*l*_** and ***σ*_*l*_** are the mean and standard deviation values at the time step *t* for the element *l* of the state vector. Please note that the use of Euler's angles is only to define trust domain since the angles are easy to interpret. However, to generate motion over *SO*(3) during the learning phase unit quaternion, interpolation is used.

## 4 Koopman Operator–Based Knowledge-Guided Learning Method

Algorithm 1. Koopman Operator–Based Knowledge- Guided DQN
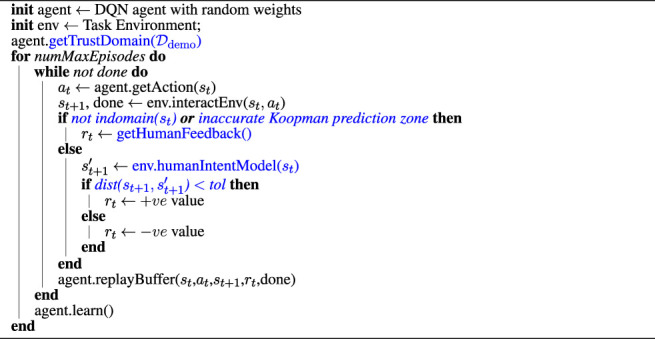


The outline of the training loop is visualized in Figure 1C, which represents a typical training loop for a DQN agent with an improved reward function block on the right-hand side of the figure. At the top of the reward function block is a decision-making block named *indomain*, which takes the agent's state after executing an action, i.e., ***s***_***t*** +1_ as input, and checks whether that state lies in the trusted domain of the Koopman prediction domain. If that block returns true, then the Koopman operator–based human intent model predicts the state for step *t* + 1, i.e., $s_{t+1}^{\prime }$ . Then, the next block invokes the *dist* method to determine whether the states ***s***_***t*** **+1**_ and $s_{t+1}^{\prime }$ are close based on Eqs. 15, 16 up to certain resolution values and returns the reward *r*_*t*+1_ as a positive number or sets it to a negative value otherwise. On the other hand, if the *indomain* block returns false, then a human expert has to manually input a positive or negative reward based on his/her evaluation.

In Algorithm 1, we present the pseudo-code for the proposed *Koopman Operator–Based Knowledge-Guided DQN* for the ease of implementation. Algorithm 1 follows the basic outline of the DQN architecture but has been improvised into learning to imitate human intended trajectories with a newly introduced reward function using the Koopman theory. The added functionalities are highlighted as blue texts. Please note, in Algorithm 1, the method *getTrustDomain* simply follows the equations described in Section 3.4. The *indomain* method is equivalent to the *indomain* block in Figure 1C, as already discussed. Furthermore, the method *getHumanFeedback* invokes a query to the human expert for a feedback in terms of positive or negative rewards. The *env.humanIntentModel* implements Eq. 13. Finally, the *dist* method is equivalent to the *dist* block of Figure 1C, which has been discussed previously. Table 1 shows the elements of the discrete action space considered in this article.

**TABLE 1:** Discrete action space with their meanings.

Action no.	Description
0	moves uniformly in position and rotation space
1	moves only in position space
2	move only in rotation space
3	moves both in position and rotation but moves more in rotation
4	moves along +x
5	moves along −x
6	moves along +y
7	moves along −y
8	moves along +z
9	moves along −z

## 5 Case Study

In this section, we provide two examples to demonstrate the utility of the proposed algorithm. The first example pertains to learning to execute *putting object in shelf* task with a universal robot (UR5 arm) from the human demonstration. The path planning for this kind of task is challenging with traditional motion planners since the motion of the end-effector is constrained to lie only in $R^{3}$ (Stilman, 2007); Sinha et al. (2021) instated SE(3) since orientation of the end-effector has to be kept constant throughout the path. The second example pertains to safely transferring a sharp-edged object to a human being. Learning to perform to plan for this type of task is challenging since in the demonstrated trajectories, both the position and orientation of the robot end-effector change, but at different rates, to ensure the safety of the human in the surroundings. Again in this example, we have used an UR5 arm to demonstrate the learning of the path planning.

### 5.1 Learning to Plan to Put an Object in Shelf

**FIGURE 2:**
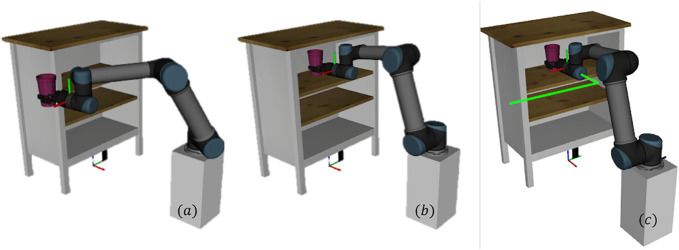
Planning to put an object in a shelf. Left: initial pose of the arm. Middle: goal pose of the arm. Right: one of the demonstrated path for *putting the object in the shelf* task.

**FIGURE 3:**
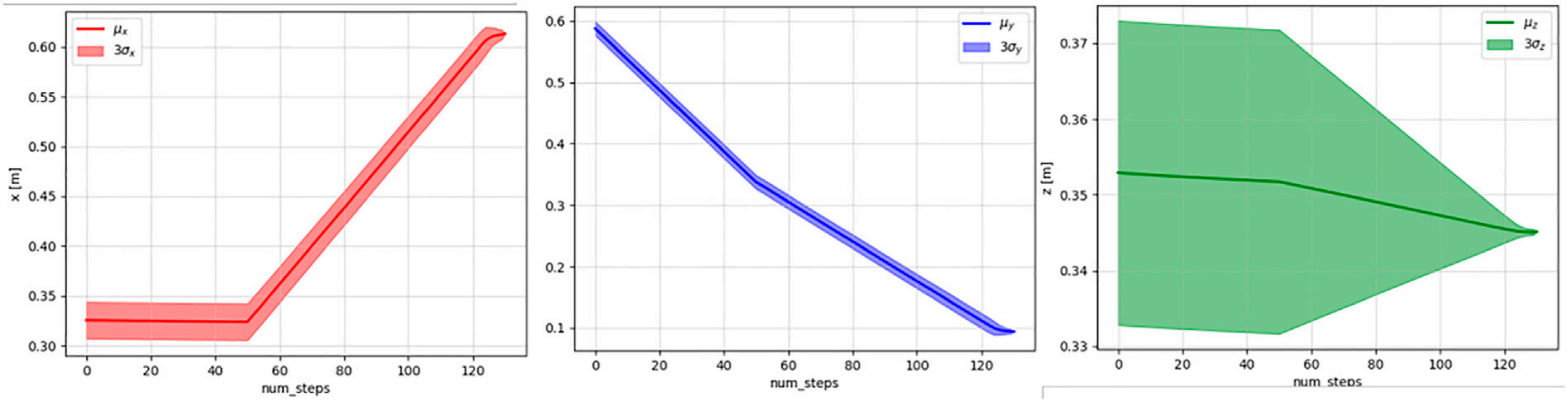
Means and three standard deviations of the *x* (left), *y* (middle), and *z* (right) values of the demonstrated trajectories.

In this example, the proposed Algorithm 1 is utilized to plan a path for putting an object into a shelf using an UR5 robot. Even for this seemingly simple task, the planning problem is challenging because of the constrained motion of the robot end-effector. More specifically, throughout the path, the end-effector's orientations are kept fixed and only the positions are changed. The learning agent has to recover that information to plan a path using the reward feedback from the human intent model developed using the demonstrated trajectories. The learning agent was successful in learning to plan a path for the task. Figure 2 shows an instance of the initial and goal end-effector poses and one of the demonstrated trajectories for this task. The distributions of the individual components of the states considering all the demonstrated trajectories are presented in the Figure 3.

Since for this particular task, the orientation of the end-effector was kept fixed, the Koopman operator–based human intent model is required only to predict the experts' end-effector position. For any action taken by the learning agent that changes the end-effector's orientation, a negative reward is provided to the learning agent. For this reason, given the end-effector poses from the demonstrated trajectories data, we extracted the position vectors $p_{t}^{i}=\left[x_{t}^{i},y_{t}^{i},z_{t}^{i}\right]$ , where $p_{t}^{i},x_{t}^{i},y_{t}^{i},z_{t}^{i}$ represent the position vector and its *x*, *y*, and *z* components at time step *t* of the *i*th demonstrated trajectory, respectively. Furthermore, we considered a second-order polynomial function $g\left({\left[a,b,c\right]}^{T}\right)={\left[a,b,c,a^{2},b^{2},c^{2},ab,ac,bc\right]}^{T}$ with the input vector [*a*, *b*, *c*], where *a*, *b*, *c* are scalars, to obtain the higher dimensional representation of the vectors $p_{t}^{i}$ s. Then, we set ***x***_***m***_’s in Eqs. 11, 12 as $g\left(p_{t}^{i}\right)$ to obtain ***K*_*p*_** using Eq. 10. This ***K*_*p*_** matrix characterizes the human intent model to predict the human preferred states.

**FIGURE 4:**
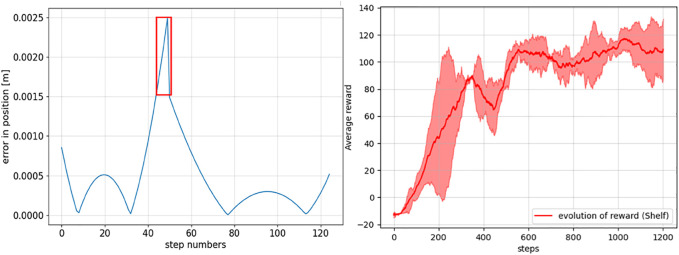
Left: absolute prediction error between the ground truth and the Koopman operator–based human intent model predicted end-effector positions at different steps. The region with a higher prediction error is marked as a red box where human intervention to provide reward is preferred. Right: evolution of the episodic cumulative rewards averaged over 100 episodes.

**FIGURE 5:**
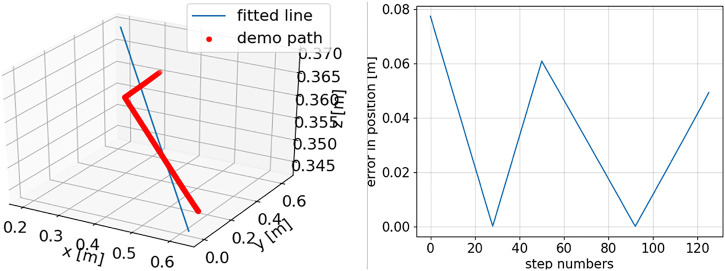
For one demonstrated trajectory: left, line approximation of the position data by minimizing the least square errors; right, line approximation errors at the different steps of the demonstrated trajectory.

The left panel of Figure 4 shows the accuracy of the trained Koopman operator to predict human preferred state $p_{t+1}^{\prime }$ given the state ***p*_*t*_** for one of the demonstrated trajectories that was not used while determining ***K*_*p*_**. It can be noticed that the predictions closely match the ground truth states (error <1.5e−3 ), indicating the quality of the learned model of the experts' demonstration at most of the steps except between step numbers 48 and 52, where the errors were comparatively higher (1.5*e* − 3 < error <2.3e−3 ), as highlighted with a red rectangular box in the left panel of Figure 4. Since in the region around 48 and 50, the Koopman operator–based human intent model has a higher prediction error, an expert intervention is requested to receive the reward after taking action by the DQN agent occasionally. The agent learned the experts' behaviour after 1,200 episodes as shown in the right panel of Figure 4, which takes ≈135 minutes on an average on a computer with Intel *i*7 processor with 16 GB memory. The structure of the deep neural network to model the DQN agent is given in Appendix 7.1 along with the parameter values for training the RL agent in Table A1. Furthermore, to compare the benefit of using the Koopman operator against a simpler fitting method, a least square line fit is performed through one of the demonstrated trajectories. As shown in the left panel of Figure 5, a line fit could not properly capture the nature of the trajectory at all. The right panel of Figure 5 shows the error between the actual demonstrated states (only positions vectors are considered since the orientation is kept fixed for this example) with respect to the fitted line. As can be noticed that the prediction error is worse in the case when the demonstrated data are modeled by fitting a line (please see right panel of Figure 5) as against that of the Koopman operator–based modeling (please see the left panel of Figure 4). This comparison demonstrates the effectiveness of the Koopman operator as a linear operator of potential complex nonlinear systems *vs*. simple linear models to capture the human intent models.

### 5.2 Learning to Safely Transfer Knife in the Close Approximation of Human

**FIGURE 6:**
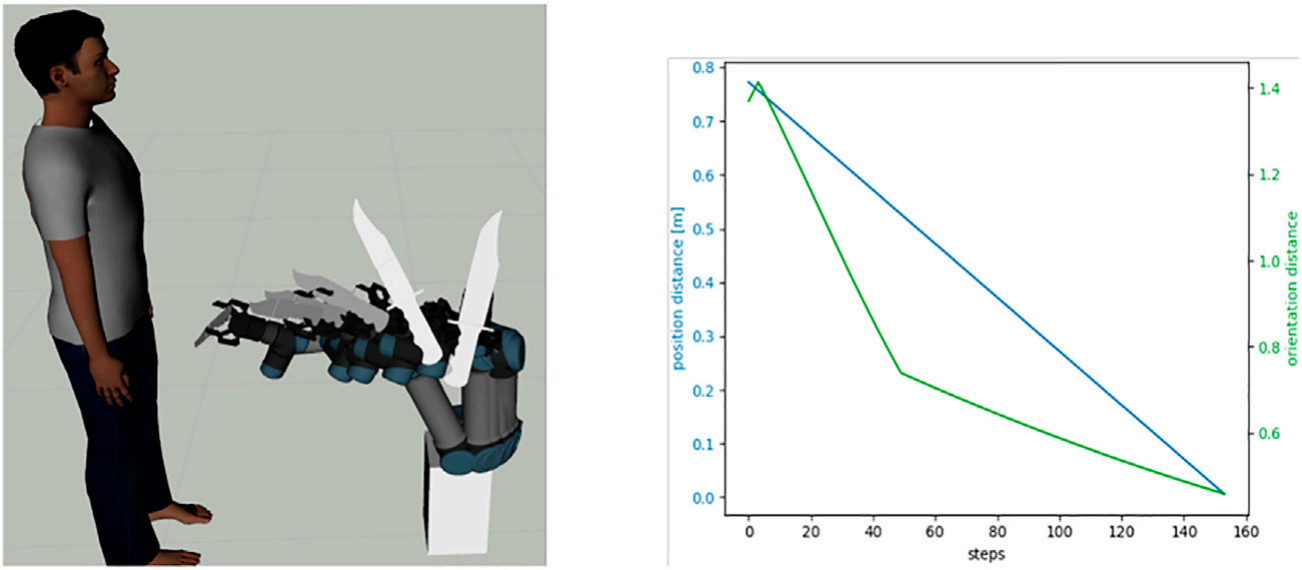
Left, the robot executing one of the demonstrated trajectories for transferring a knife safely to a human. Right: characteristic of the change of the positional (blue) and orientational (green) distance to the goal at different steps of one of the demonstrated paths.

The objective of this example is to utilize Algorithm 1 to make the DQN agent learn to transfer a sharp object (e.g., a knife) safely while a human being is in close proximity of an UR5 robot. The initial pose of the robot is such that the sharp edge of the knife is held upward which is an unsafe pose to deliver the object to a human being (see left panel of Figure 6). Ideally, the knife should be transferred in such a way such that the sharp edge is brought down as quickly as possible before transferring the sharp-edged object to the human. Also, at the goal pose when the knife reaches in front of the human, the sharp edge should be completely in the downward direction. The left panel of Figure 6 shows the initial, goal, and some of the intermediate poses of the end-effector for one of the demonstrated trajectories. This task is particularly interesting because throughout the path, both the position and orientation of the end-effector change during the execution of the task while the rotational distance to the goal is minimized faster than that of the positional distance. The changes in position and orientation distances from the start to the goal pose, are shown in the right panel of Figure 6, respectively. It can be noted that throughout the steps of the demonstrated path, the position distance was reduced at the same rate, whereas the orientation distance was reduced at a higher rate in the initial steps and at a lower rate afterward to ensure the knife edge is brought downward rapidly before passing it to the human.

**FIGURE 7:**
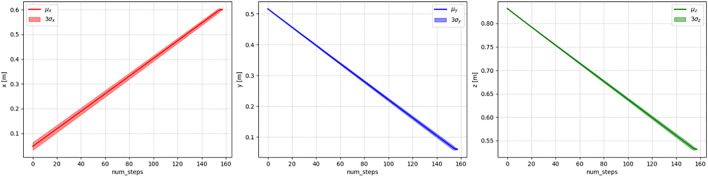
Means and three standard deviations of the *x* (left), *y* (middle), and *z* (right) values of the demonstrated trajectories.

**FIGURE 8:**
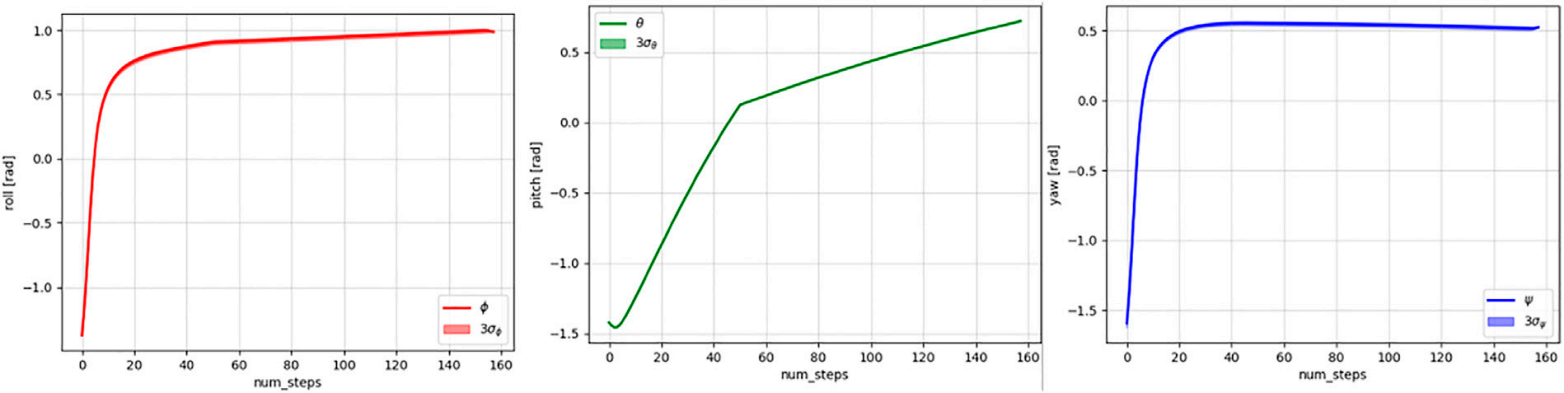
Means and three standard deviations of the *roll* (left), *pitch* (middle), and *yaw* (right) values of the demonstrated trajectories.

In this example, the planning agent has to learn the expert's trajectory where the end-effector's position and orientation both change and at different rates at different segments of the trajectory. The span of the demonstrated trajectories used to obtain the human knowledge representation using the Koopman operator is shown in Figures 7, 8, respectively. Furthermore, since in this task, both the position and orientation of the robot's end-effector change, the Koopman operator–based human intent model is characterized by two matrices ***K*_*p*_** and ***K*_*o*_**, such that

$$g\left(p_{t+1}^{\prime }\right)=K_{p}g\left(p_{t}\right)$$ (17)

$$g\left(o_{t+1}^{\prime }\right)=K_{o}g\left(o_{t}\right)$$ (18)

where $p_{t}={\left[x_{t},y_{t},z_{t}\right]}^{T}\in R^{3},o_{t}={\left[\alpha_{t},\beta_{t},\gamma_{t}\right]}^{T}\in R^{3}$ are the position and orientation vectors of the learning agent at time step *t*, *x*_*t*_, *y*_*t*_, *z*_*t*_ are the components of ***p*_*t*_**, and *α*_*t*_, *β*_*t*_, *γ*_*t*_ are the roll, pitch, and yaw angles representing the components of ***o*_*t*_**, respectively. Also, the function $g\left(\cdot \right)\in R^{9}$ is defined as before. Therefore, both ***K*_*p*_** and $K_{o}\;\in R^{9\times 9}$ as per Eqs 17 and 18.

In order to obtain ***K*_*p*_** and ***K*_*o*_**, we have used Eq. 10 with $x_{m}=g\left(p_{t}^{i}\right)$ and $x_{m}=g\left(o_{t}^{i}\right)$ , respectively, where $p_{t}^{i}$ and $o_{t}^{i}$ are the position and orientation vectors of the way point of the *i*th demonstrated trajectory at time step *t*. Please note that we could have concatenated $p_{t}^{i}$ and $o_{t}^{i}$ and used $g^{\prime }\left({\left[p_{t}^{i},o_{t}^{i}\right]}^{T}\right)$ to learn a single ***K*** matrix to characterize the Koopman operator–based human intent model. However, in such a case, the size of the predicted vector would be bigger, which will become prone to greater residual error than that of a smaller predicted vector. In this case, ***g*** **′**(⋅) would be a function that takes a vector of six elements $\left({\left[p_{t}^{i},o_{t}^{i}\right]}^{T}\in R^{6}\right)$ and returns a second-order polynomial with the components of the input vector.

**FIGURE 9:**
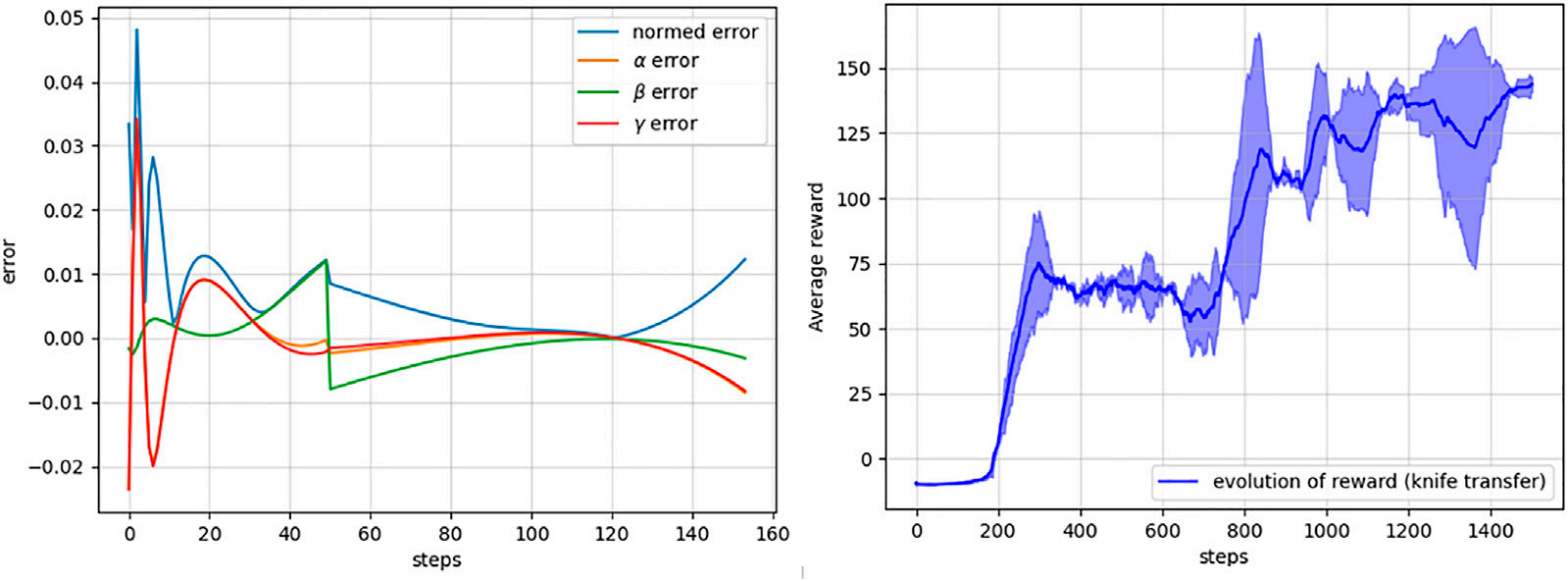
Left: prediction errors of the learned Koopman operator for the orientation elements, *α*, *β*, *γ*, or roll, pitch, and yaw elements with respect to the ground truth over different steps. Right: mean and standard deviation of the cumulative rewards over the episodes during the training of the DRL agent for the knife transfer task obtained with multiple trial runs of the learning task.

Once the matrices ***K*_*p*_** and ***K*_*o*_** are identified, for any given position and orientation vectors of the learning agent at time step *t*, the human preferred states can be predicted using Eqs. 17, 18, respectively. To test the performance of the learned human intent model, we took the states of one of the demonstrated trajectories and predicted the next position and orientation states using ***K*_*p*_** and ***K*_*o*_**, respectively. This trajectory was not used while identifying ***K*_*p*_** and ***K*_*o*_**. We found that the maximum predicted position error was in the order of submillimeters and did not vary much for different steps. However, the prediction errors of the orientation states varied over the step as shown in the left panel of Figure 9.

In the right panel of Figure 9, we present the evolution of the accumulated reward of the DQN agent while learning the task using the proposed Algorithm 1. The learning agent took ≈210 minutes on average to learn the task. Please refer to Appendices 7.1 and 7.2 for more information about the structure of the neural network used to model the learning agent and the other hyper-parameters used for this task, respectively.

## 6 Conclusion

In this article, we have proposed a novel knowledge-guided RL framework for arm-type robots to learn from expert demonstrated trajectories. This is achieved by developing a human intent model based on the Koopman operator theory utilizing the data from the human demonstrated trajectories. This Koopman operator–based human intent model is then used to shape a reward function for a DQN agent which should act as an autonomous planner upon successful training. Furthermore, we have used the span of the coordinates of the poses of the demonstrated trajectories to help the learning agent to decide whether to rely on the Koopman operator–based human knowledge representation prediction or not such that the agent does not receive a spurious reward during its learning stage which would otherwise hinder the learning process. We have presented two examples that utilize our proposed Koopman operator–based knowledge-guided RL algorithm to learn the tasks satisfying human intent. The second task not only shows the efficacy of the proposed algorithm but also demonstrates how the algorithm can be used to add safety measures in performing a task while humans are in close proximity.

This work paves the way for many future research directions to be pursued. In this work, we have considered a discrete action space which is a limitation that we want to work on in the future to make the algorithm work in more general settings by utilizing the continuous action space. Furthermore, we have assumed that there are no obstacles around the robots' workspace. It would be interesting to extend this work to environments with obstacles.

## Data Availability

The raw data supporting the conclusions of this article will be made available by the authors, without undue reservation.

## 7 Appendix

### 7.1 Deep Q-Learning Structure

The learning agent and target agent both have the same structure with two hidden layers each having 1,024 units of neurons for the putting object in shelf task (task 1) or 1,560 units for knife transfer task (task 2) and *relu* activation for both task 1 and task 2. In the current implementation, the agent can observe the full end-effector pose and its distance to the goal, both in position and orientation spaces. Hence, the dimension of the observed state is 8 (pose: 6, position distance to goal: 1, and orientation distance to goal: 1). The input layer for task 1 has the dimension 5 (3 for position coordinates and 2 for the position and orientation distances). For task 2, the dimension of the input layer is 8 (3 for position coordinates, 3 for rotation coordinates, and 2 for position and orientation distances). The output of the DQN has the same dimension as that of the size of the action space which is 10 for both of the tasks considered in this article. Each element of the output vector represents the *Q* value of the state–action pair. The neural network is compiled with *ADAM* optimizer and *MSE* loss. We have used TensorFlow version 2.7 to model the neural network.

### 7.2 Hyper-Parameters

**TABLE A1:** Hyper-parameters used in the DRL training process.

Parameter description	Task 1	Task 2
Max. number of episodes	1,500	1,200
Tolerance to reach goal	0.005	0.005
Tolerance to compare poses	0.002	0.002
DQN agents' learning rate	1e-5	1e-4
Size of the replay buffer	10,000	10,000
Batch size	1,000	1,000

## References

[B1] Abraham I. De La Torre G. Murphey T. D. (2017). “Model-based Control Using Koopman Operators,” in 2017 Robotics: Science and Systems, RSS 2017 (MIT Press Journals). 10.15607/rss.2017.xiii.052

[B2] Abraham I. Murphey T. D. (2019). Active Learning of Dynamics for Data-Driven Control Using Koopman Operators. IEEE Trans. Robot. 35, 1071–1083. 10.1109/tro.2019.2923880

[B3] Bakker P. Kuniyoshi Y. (1996). “Robot See, Robot Do: An Overview of Robot Imitation,” in AISB96 Workshop on Learning in Robots and Animals (Springer), 3–11.

[B4] Billard A. Matarić M. J. (2001). Learning Human Arm Movements by Imitation:. Robotics Aut. Syst. 37, 145–160. 10.1016/s0921-8890(01)00155-5

[B5] Broad A. Abraham I. Murphey T. Argall B. (2020). Data-driven Koopman Operators for Model-Based Shared Control of Human-Machine Systems. Int. J. Robotics Res. 39, 1178–1195. 10.1177/0278364920921935

[B6] Chernova S. Veloso M. (2008). “Teaching Collaborative Multi-Robot Tasks through Demonstration,” in Humanoids 2008-8th IEEE-RAS International Conference on Humanoid Robots (IEEE), 385–390. 10.1109/ichr.2008.4755982

[B7] Christiano P. Leike J. Brown T. B. Martic M. Legg S. Amodei D. (2017). Deep Reinforcement Learning from Human Preferences. arXiv preprint arXiv:1706.03741.

[B8] Finn C. Levine S. Abbeel P. (2016). “Guided Cost Learning: Deep Inverse Optimal Control via Policy Optimization,” in International Conference on Machine Learning (Berkeley, Berkeley, USA: University of California), 49–58.

[B9] Gao X. Si J. Wen Y. Li M. Huang H. H. (2020). “Knowledge-guided Reinforcement Learning Control for Robotic Lower Limb Prosthesis,” in 2020 IEEE International Conference on Robotics and Automation (ICRA) (IEEE), 754–760. 10.1109/icra40945.2020.9196749

[B10] Griffith S. Subramanian K. Scholz J. Isbell C. L. Thomaz A. L. (2013). Policy Shaping: Integrating Human Feedback with Reinforcement Learning. Georgia Institute of Technology.

[B11] Henrion D. Mezic I. Putinar M. (2016). Applied Koopmanism. arXiv:1206.3164.

[B12] Hester T. Vecerik M. Pietquin O. Lanctot M. Schaul T. Piot B. (2018). “Deep Q-Learning from Demonstrations,” in Proceedings of the AAAI Conference on Artificial Intelligence (AAAI).

[B13] Jin W. Murphey T. D. Kulić D. Ezer N. Mou S. (2020). Learning from Sparse Demonstrations. arXiv preprint arXiv:2008.02159.

[B14] LaValle S. M. (2006). Planning Algorithms. Cambridge University Press.

[B15] Lusch B. Kutz J. N. Brunton S. L. (2018). Deep Learning for Universal Linear Embeddings of Nonlinear Dynamics. Nat. Commun. 9 (1), 1–10. 10.1038/s41467-018-07210-0 30470743 PMC6251871

[B16] Ng A. Y. Russell S. J. (2000). Algorithms for Inverse Reinforcement Learning. Icml 1, 2.

[B17] Niekum S. Osentoski S. Konidaris G. Chitta S. Marthi B. Barto A. G. (2015). Learning Grounded Finite-State Representations from Unstructured Demonstrations. Int. J. Robotics Res. 34, 131–157. 10.1177/0278364914554471

[B18] Reddy S. Dragan A. D. Levine S. (2019). Sqil: Imitation Learning via Reinforcement Learning with Sparse Rewards. arXiv preprint arXiv:1905.11108.

[B19] Ross S. Bagnell D. (2010). “Efficient Reductions for Imitation Learning,” in Proceedings of the Thirteenth International Conference on Artificial Intelligence and Statistics (JMLR Workshop and Conference Proceedings), 661–668.

[B20] Ross S. Bagnell J. A. (2014). Reinforcement and Imitation Learning via Interactive No-Regret Learning. arXiv preprint arXiv:1406.5979.

[B21] Ross S. Gordon G. Bagnell D. (2011). “A Reduction of Imitation Learning and Structured Prediction to No-Regret Online Learning,” in Proceedings of the Fourteenth International Conference on Artificial Intelligence and Statistics (JMLR Workshop and Conference Proceedings), 627–635.

[B22] Schaal S. (1997). Learning from Demonstration. Adv. neural Inf. Process. Syst. 1, 1040–1046.

[B23] Schaal S. (1999). Is Imitation Learning the Route to Humanoid Robots? Trends cognitive Sci. 3, 233–242. 10.1016/s1364-6613(99)01327-3 10354577

[B24] Sinha A. Sarker A. Chakraborty N. (2021). “Task Space Planning with Complementarity Constraint-Based Obstacle Avoidance,” in International Design Engineering Technical Conferences and Computers and Information in Engineering Conference. Vol. Volume 8B: 45th Mechanisms and Robotics Conference (MR) (ASME). 10.1115/detc2021-72009

[B25] Stilman M. (2007). “Task Constrained Motion Planning in Robot Joint Space,” in 2007 IEEE/RSJ International Conference on Intelligent Robots and Systems (IEEE), 3074–3081. 10.1109/iros.2007.4399305

[B26] Torabi F. Warnell G. Stone P. (2018). Behavioral Cloning from Observation. arXiv preprint arXiv:1805.01954.

[B27] Van Hasselt H. Guez A. Silver D. (2016). “Deep Reinforcement Learning with Double Q-Learning,” in Proceedings of the AAAI Conference on Artificial Intelligence. Vol. 30 (AAAI).

[B28] Williams M. O. Kevrekidis I. G. Rowley C. W. (2015). A Data-Driven Approximation of the Koopman Operator: Extending Dynamic Mode Decomposition. J. Nonlinear Sci. 25, 1307–1346. 10.1007/s00332-015-9258-5

[B29] Wirth C. Akrour R. Neumann G. Fürnkranz J. (2017). A Survey of Preference-Based Reinforcement Learning Methods. J. Mach. Learn. Res. 18, 1–46.

[B30] Ziebart B. D. Maas A. L. Bagnell J. A. Dey A. K. (2008). Maximum Entropy Inverse Reinforcement Learning. AAAI (Chicago,IL,USA) 8, 1433–1438.

